# Association of antidiabetic therapies with lower extremity amputation, mortality and healthcare cost from a nationwide retrospective cohort study in Taiwan

**DOI:** 10.1038/s41598-021-86516-4

**Published:** 2021-03-26

**Authors:** Hsien-Yen Chang, Ying-Yi Chou, Wenze Tang, Guann-Ming Chang, Chi‐Feng Hsieh, Sonal Singh, Yu-Chi Tung

**Affiliations:** 1 Department of Health Policy and Management, Johns Hopkins Bloomberg School of Public Health, Baltimore, MD USA; 2 Center for Population Health IT, Johns Hopkins Bloomberg School of Public Health, Baltimore, MD USA; 3 Center for Drug Safety and Effectiveness, Johns Hopkins Bloomberg School of Public Health, Baltimore, MD USA; 4 Institute of Health Policy and Management, School of Public Health, National Taiwan University, Room 634, No.17, Xu-Zhou Road, Taipei, 100 Taiwan; 5 Department of Epidemiology, Harvard School of Public Health, Boston, MA USA; 6 Department of Family Medicine, Chang-Gung Memorial Hospital, Taoyuan, Taiwan; 7 School of Medicine for International Students, I- Shou University, Kaohsiung, Taiwan; 8 Department of Family Medicine and Community Health, University of Massachusetts Medical School, Worcester, MA USA

**Keywords:** Type 2 diabetes, Health care economics, Outcomes research, Adverse effects

## Abstract

We compared risks of clinical outcomes, mortality and healthcare costs among new users of different classes of anti-diabetic medications. This is a population-based, retrospective, new-user design cohort study using the Taiwan National Health Insurance Database between May 2, 2015 and September 30, 2017. An individual was assigned to a medication group based on the first anti-diabetic prescription on or after May 1, 2016: SGLT-2 inhibitors, DPP-4 inhibitors, GLP-1 agonists or older agents (metformin, etc.). Clinical outcomes included lower extremity amputation, peripheral vascular disease, critical limb ischemia, osteomyelitis, and ulcer. We built three Cox proportional hazards models for clinical outcomes and mortality, and three regression models with a log-link function and gamma distribution for healthcare costs, all with propensity-score weighting and covariates. We identified 1,222,436 eligible individuals. After adjustment, new users of SGLT-2 inhibitors were associated with 73% lower mortality compared to those of DPP-4 inhibitors or users of older agents, while 36% lower total costs against those of GLP-1 agonists. However, there was no statistically significant difference in the risk of lower extremity amputation across medication groups. Our study suggested that SGLT-2 inhibitors is associated with lower mortality compared to DPP 4 inhibitors and lower costs compared to GLP-1 agonists.

## Introduction

As of 2015, 23.1 million Americans of all ages have been diagnosed with diabetes^1^. An estimated 1.5 million U.S. adults aged 18 years or older were additionally diagnosed each year^2^. In order to manage blood glucose level and prevent long-term macrovascular and microvascular complications, diabetes medications are often prescribed apart from necessary lifestyle modification. These medications can be classified into older agents such as biguanides (e.g. metformin), sulfonylureas and thiazolidinediones (TZD) and newer agents including sodium-glucose cotransporter 2 (SGLT-2) inhibitors, glucagon-like peptide 1 (GLP-1) agonists and dipeptidyl peptidase 4 (DPP-4) inhibitors. Compared to some other agents, the SGLT-2 inhibitors work by inhibiting the re-absorption of glucose in the kidney.

The first drug under the agent class SGLT-2 inhibitors, canagliflozin, was approved by US Food and Drug Administration (FDA) in 2013, followed by the approval of two other drugs under the same class, dapagliflozin and empagliflozin. Subsequent observational studies have yielded inconsistent results regarding risk of death and relative cost associated with SGLT-2 inhibitors^3–10^. However, such studies (1) only compared SGLT-2 inhibitors to a single class of other anti-diabetes medication or a reference group that consists of multiple classes of agents, and (2) only investigated into either clinical outcome(s) or cost outcome(s)^4,5,9,10^. Some of the studies also suffer from the methodological limitations such as treating switchers as new users^11,12^ and potential insufficient confounding adjustment^13,14^. To address these limitations, we evaluated the association of each class of anti-diabetes medications to various outcomes including all-cause mortality, clinical outcomes (including lower-extremity amputation), and costs within the same population.

## Methods

### Study design and data source

We conducted a population-based, retrospective, new-user design cohort study using the Taiwan National Health Insurance Research Database (NHIRD) between May 2, 2015 and September 30, 2017 (the study period) to investigate the association between anti-diabetic medications and three types of outcomes (clinical outcomes, death and daily healthcare costs). NHIRD, provided by the National Health Insurance Administration (NHIA), consists of individual-level healthcare usage data and includes the following individual files: inpatient files (including diagnosis codes, procedure codes, medication codes, date of admission and discharge, and costs), ambulatory files (including diagnosis codes, procedure codes, date of visit, and costs), pharmaceutical files (including medication codes, date of prescription/fill, days of supply, and costs), and beneficiary files (including sex, date of birth, and date of death). The Research Ethics Committee of the National Taiwan University Hospital approved the study. The requirement of informed consent was waived because the dataset we used in this study was deidentified secondary data.

### Cohort Derivation

**Figure 1:**
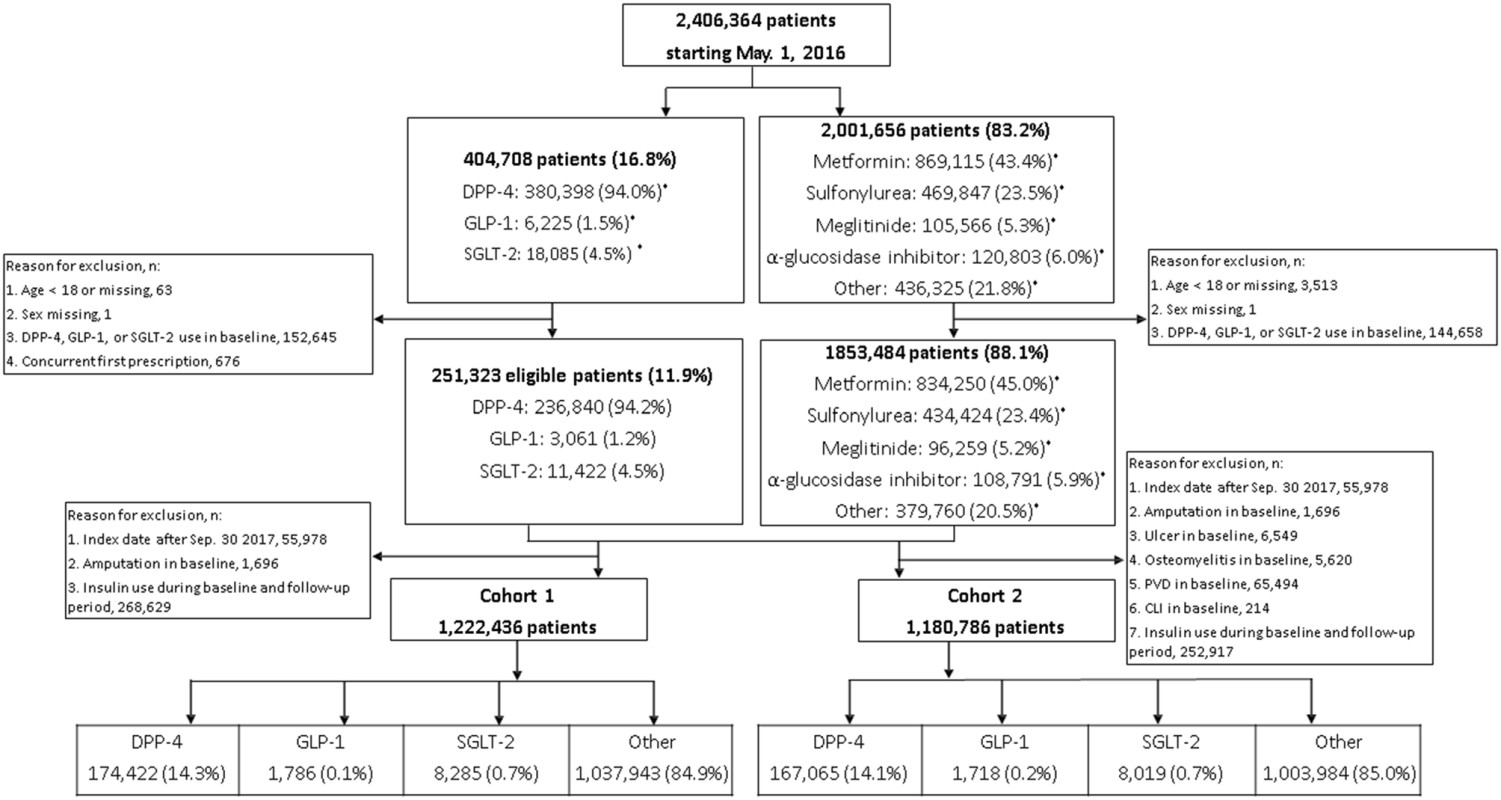
Flowchart of the sample. CLI, critical limb ischemia; DPP-4, dipeptidyl peptidase 4 inhibitors; GLP-1, glucagon-like peptide 1 analogues; PVD, peripheral vascular disease; SGLT-2, sodium-glucose cotransporter 2 inhibitors. *Patients.

Patients were included in this study if they had the following anti-diabetic medications records: SGLT-2 inhibitors, DPP-4 inhibitors, GLP-1 agonists, or older agents (metformin, sulfonylureas, combinations of oral blood glucose lowering drugs, alpha glucosidase inhibitors, thiazolidinediones, or other blood glucose lowering drugs). We used anatomical therapeutic chemical (ATC) code to identify these medications. Figure 1 depicts the process of selecting study subjects. Regarding patients with SGLT-2 inhibitors, DPP-4 inhibitors, or GLP-1 agonists, the starting date of the observation (the index date) for patients with *any* newer agent was the first prescription date of such medication on or after May 1, 2016 because it was the first date when these new drugs were all available (new user design). Under Taiwan’s National Health Insurance (NHI), SGLT-2 inhibitors were reimbursed on May 1, 2016, DPP-4 inhibitors were on March 1, 2009, and GLP-1 inhibitors were on February 1, 2015. Regarding patients with only older agents, the index date for patients with any older agent during the study period was the first prescription date of such medication on or after May 1, 2016. For clinical outcomes and death, the last date of observation was defined as the earliest of the following five dates^3^: (1) the last date with the continuous supply of the index medication at hand plus a grace period of 30 days to account for the drug clearance^15^; (2) the date before the use of other newer agents (for patients with any new agent); (3) the death date; (4) the study end date, September 30, 2017; or (5) the first date of the outcome; patients whose last observation date not equal to the first date of a specific outcome were censored for that given outcome. For daily healthcare costs, the last date of observation would be the earliest of the following dates: (1) the last date with the continuous supply of the index medication at hand plus 30 days; (2) the date prior to the use of other newer agents; (3) the death date; or (4) the study end date, September 30, 2017. The length of observation time (days) was calculated as the last date minus the first date plus 1 day.

From 23 million Taiwanese nationals enrolled in Taiwan’s National Health Insurance (NHI), we identified 2,406,364 patients with anti-diabetic medications. Then, we excluded patients if they met the following criteria: younger than 18 years old on the index date; unknown gender; using any newer anti-diabetic medication during the baseline period, a 365-day period prior to the index date; using insulin products during the entire study period; having records of two or more newer agents on the first prescription for patients with *any* newer agent; having an index date after September 30, 2017; and experiencing an outcome(s) of interest during the baseline period. Because amputation was rare and we wanted to include as many patients as possible, we derived two separate cohorts: the first cohort included patients without baseline amputation (n = 1,222,436) and the second cohort included patients without any baseline clinical outcome (n = 1,180,786). The first cohort was used for analyses of outcomes including amputation, all-cause mortality and healthcare costs; the second cohort was used for the remaining four clinical outcomes (peripheral vascular disease, critical limb ischemia, osteomyelitis, and ulcer).

### Definition of exposure

Patients with any newer medication between May 1, 2016 and September 30, 2017 were assigned to the corresponding medication group (SGLT-2 inhibitors, DPP-4 inhibitors, or GLP-1 agonists) based on the first prescription of the newer medications; the remaining patients with only older agents were assigned to a fourth group (“first-line agent”). Patients assigned to newer medication groups might be prescribed first-line medications in the baseline period, or we would not have enough patients for this study^3^.

### Definition of outcomes

We included three types of outcomes: clinical outcomes, death and daily healthcare costs. Clinical outcomes included foot and leg amputation, ulcer, osteomyelitis, peripheral vascular disease, and critical limb ischemia; they were defined by administrative codes based on literature review^3,16–21^. Detailed definitions can be found in the Supplementary Appendix. Both inpatient and outpatient diagnosis codes were used for outcome definitions. Date of death was obtained from the beneficiary files^9,12,13^. We included three types of healthcare costs: total costs (including both medical and pharmacy costs), emergency costs, and inpatient costs^14^. We summed up the costs incurred over the observation period and divided the amount by the length of observation measured in days to derive daily costs by types. Under Taiwan’s NHI, healthcare products and services are reimbursed by a floating-point-value mechanism, which adjusts the point value to ensure the total amount of payments would not exceed the annual global budget. Based on the fluctuating point mechanism, 1 point is usually adjusted to slightly less than one New Taiwan Dollar (NTD); we assumed that 1 point was equal to 1 NTD for easier interpretation^22^.

### Definition of confounders

Through literature review, we included the following confounders: demographics (gender and age), diabetes severity, comorbidities and medication histories during the baseline period. We used the adapted Diabetes Complications Severity Index score (aDCSI) to measure a patient’s diabetes severity. The aDCSI is composed of a score of 7 diabetes-related complications weighted by severity; the score increases as the severity increases^23,24^. For comorbidities, we calculated the following medical conditions: cerebrovascular disease, congestive heart failure, ischemic heart disease, hypertension, retinopathy, nephropathy, neuropathy, atrial fibrillation, renal disease, and eye disease^3^. We also assessed the medication utilization, including angiotensin-converting enzyme inhibitors, anticoagulants, angiotensin receptor blockers, aspirin, antiasthmatic drugs, bile acid sequestrants, carbonic anhydrase inhibitors, calcium channel blockers, fibrates, hormone replacement therapy, loop diuretic, β-blockers, platelet aggregation inhibitors, potassium-sparing diuretic, statins, and thiazides^3^.

### Propensity score

In order to ensure the balance of confounding between the SGLT-2 inhibitor group and other three groups, 3 sets of propensity score-based weights were developed separately for 2 cohorts^3,25–27^. The logistic regression model, including aforementioned confounders, was used to determine the likelihood of becoming a SGLT-2 inhibitor user among new users of SGLT-2 inhibitors and: (1) new users of DPP-4 inhibitors, (2) new users of GLP-1agonists, and (3) users of older agents.

Various methods have been used to apply propensity scores; however, each method has its advantages and disadvantages^28,29^. We adopted propensity score weighting because we wanted to estimate one interpretable overall treatment effect and we did not want to lose any observation. The average treatment effect of the treated (ATT) weighting was applied to estimate the average treatment effect on new users of SGLT-2 inhibitors; that is, we compared risk-adjusted outcomes among new SGLT-2 inhibitor users with the hypothesized situation had they taken DPP-4 inhibitors, GLP-1 agonists or other first-line agents instead of SGLT-2 inhibitors. This approach is useful when systematic differences may exist between the study sample and the overall population^30^. The standardized difference was calculated to compare the balance in the baseline confounders before and after ATT weighting; a standardized difference less than 0.1 was considered to be negligible^30^.

### Statistical analysis

Chi-square tests for categorical variables and Kruskal–Wallis tests for continuous variables were used to assess the differences of patients’ characteristics among 4 medication groups. For each clinical outcome and death, we developed 3 separate Cox proportional hazards regression models with ATT weighting (with or without potential confounders). For each type of daily costs, we also constructed 3 multivariate linear regression models with a log-link function, gamma distribution and ATT weighting (with or without potential confounders)^31,32^. The gamma distribution was adapted because health care costs usually exhibited non-negative and positively skewed distribution; besides, it can account for the higher proportion of people with very high costs^31^. The log-link function is related to a proportional change in mean costs^31,32^. We used SAS Enterprise Guide 7.1 for all analyses. All statistical tests were two-tailed and used a type I error rate of 0.05.

## Results

### Patient characteristics

Table 1 shows the characteristics of the study subjects from the first cohort and demonstrates there were significant differences in all covariates among 4 groups of medication users. New users of SGLT-2 inhibitors were younger (56.2 vs 65.3 years) and more unlikely to have severe diabetes (1.1 vs 1.4 scores) and comorbidities of cerebrovascular disease (7.0% vs 12.8%), hypertension (65.6% vs 72.7%), nephropathy (8.2% vs 16.9%), and renal disease (8.4% vs 17.6%) compared with new users of DPP-4 inhibitors. New users of SGLT-2 inhibitors were more likely to be male (53.1% v 45.0%) compared with new users of GLP-1 inhibitors. New users of SGLT-2 inhibitors were younger (56.2 vs 61.9 years), and more likely to be male (53.1% v 51.6%) and have severe diabetes (1.1 vs 0.8 score) and comorbidities of eye disease (11.4% vs 6.6%) compared with users of first-line medications. After ATT weighting, the standardized differences of all confounders were reduced to 0.03 or smaller in Supplementary Table S1, which suggested that the balance has been achieved between the SGLT-2 inhibitor group and other three groups.

**Table 1:** Characteristics of the study sample from first cohort.

Characteristic	SGLT-2 (n = 8,285)		DPP-4 (n = 174,422)		GLP-1 (n = 1,786)		Other (n = 1,037,943)		*P*
Female, No. (%)	3,884	46.9	82,015	47.0	982	55.0	501,876	48.4	< 0.001
Age, mean (SD)	56.2	12.0	65.3	11.8	53.6	12.1	61.9	12.8	< 0.001
Age, No. (%)									
18–34 years	368	4.4	1,346	0.8	110	6.2	28,155	2.7	< 0.001
35–44 years	1,112	13.4	6,645	3.8	340	19.0	69,483	6.7	
45–54 years	2,098	25.3	24,135	13.8	507	28.4	185,397	17.9	
55–64 years	2,796	33.7	54,056	31.0	510	28.6	332,871	32.1	
≧ 65 years	1,911	23.2	88,240	50.6	319	17.8	422,037	40.6	
aDCSI score, mean (SD)	1.1	1.2	1.4	1.5	1.2	1.3	0.8	1.2	< 0.001
aDCSI score, No. (%)									
0	3,456	41.7	59,549	34.1	696	39.0	553,897	53.4	< 0.001
1	2,314	27.9	44,398	25.5	507	28.4	239,794	23.1	
2	1,527	18.4	36,029	20.7	298	16.7	150,027	14.5	
≧ 3	988	12.0	34,446	19.7	285	15.9	94,225	9.0	
Baseline use of antidiabetes medication, No. (%)									
Biguanides (Metformin)	1,455	17.6	15,709	9.0	201	11.3	245,536	23.7	< 0.001
Sulfonylureas	1,318	15.9	15,871	9.1	184	10.3	144,052	13.9	< 0.001
Meglitinide	830	10.0	7,327	4.2	85	4.8	37,777	3.6	< 0.001
α-glucosidase inhibitor	797	9.6	10,215	5.9	110	6.2	43,155	4.2	< 0.001
Combinations	1,933	23.3	9,900	5.7	183	10.2	103,415	10.0	< 0.001
Other drugs	185	2.2	4,725	2.7	51	2.9	20,570	2.0	< 0.001
Baseline comorbidities, No. (%)									
Cerebrovascular disease	581	7.0	22,363	12.8	103	5.8	101,288	9.8	< 0.001
Congestive heart failure	468	5.6	11,821	6.8	71	4.0	43,987	4.2	< 0.001
Ischemic heart disease	1,505	18.2	37,589	21.6	235	13.2	157,901	15.2	< 0.001
Hypertension	5,438	65.6	126,786	72.7	1,191	66.7	685,512	66.0	< 0.001
Retinopathy	1,128	13.6	29,687	17.0	324	18.1	94,656	9.1	< 0.001
Nephropathy	677	8.2	29,443	16.9	225	12.6	76,416	7.4	< 0.001
Neuropathy	513	6.2	14,520	8.3	137	7.7	49,740	4.8	< 0.001
Atrial fibrillation	133	1.6	4,235	2.4	23	1.3	17,235	1.7	< 0.001
Renal disease	697	8.4	30,690	17.6	227	12.7	81,048	7.8	< 0.001
Eye disease	942	11.4	22,699	13.0	272	15.2	68,405	6.6	< 0.001
Baseline medications, No. (%)									
ACE inhibitors	291	3.5	4,828	2.8	45	2.5	36,159	3.5	< 0.001
Anticoagulants	841	10.2	16,211	9.3	132	7.4	111,157	10.7	< 0.001
Angiotensin receptor blockers	1,110	13.4	18,571	10.6	194	10.9	137,381	13.2	< 0.001
Aspirin	744	9.0	14,065	8.1	114	6.4	99,042	9.5	< 0.001
Asthma	3,228	39.0	62,326	35.7	730	40.9	394,948	38.1	< 0.001
Bile acid sequestrants	1	0.0	44	0.0	0	0.0	195	0.0	0.290
Carbonic anhydrase inhibitors	133	1.6	3,785	2.2	32	1.8	15,946	1.5	< 0.001
Calcium channel blockers	1,110	13.4	22,414	12.9	185	10.4	169,550	16.3	< 0.001
Fibrates	593	7.2	10,717	6.1	79	4.4	73,390	7.1	< 0.001
Hormone replacement therapy	262	3.2	3,173	1.8	84	4.7	35,487	3.4	< 0.001
Loop diuretic	474	5.7	11,675	6.7	91	5.1	49,721	4.8	< 0.001
β-Blockers	1,045	12.6	16,225	9.3	164	9.2	127,237	12.3	< 0.001
Platelet aggregation inhibitors	1,593	19.2	30,242	17.3	285	16.0	219,181	21.1	< 0.001
Potassium-sparing diuretic	182	2.2	3,090	1.8	28	1.6	15,185	1.5	< 0.001
Statin	2,016	24.3	31,932	18.3	274	15.3	265,112	25.5	< 0.001
Thiazide	308	3.7	6,206	3.6	50	2.8	38,448	3.7	0.005

ACE, angiotensin-converting enzyme; aDCSI, adapted Diabetes Complications Severity Index; DPP-4, dipeptidyl peptidase 4; GLP-1, glucagon-like peptide 1; SD, standard deviation; SGLT-2, sodium-glucose cotransporter 2.

In addition, new users of SGLT-2 inhibitors (statin prescriptions: 24.3%, angiotensin receptor blockers: 13.4%, anticoagulants: 10.2%, Calcium channel blockers: 13.4%, β-Blockers: 12.6%) were more likely to have statin prescriptions, angiotensin receptor blockers , anticoagulants, Calcium channel blockers, andβ-Blockers than those using DPP-4 inhibitors (statin prescriptions:18.3%, angiotensin receptor blockers: 10.6%, anticoagulants: 9.3%, Calcium channel blockers: 12.9%, β-Blockers: 9.3%) and GLP-1 agonists (statin prescriptions:15.3%, anticoagulants: 7.4%, Calcium channel blockers: 10.4%, β-Blockers: 9.2%). Use of Platelet aggregation inhibitors at baseline is more prevalent among new users of SGLT-2 inhibitor (19.2%) compared to new users of DPP-4 inhibitors (17.3%), or GLP-1 agonists (16.0%).

Characteristics of the study sample from the second cohort where patients were required not to have had any of five clinical outcomes was presented in Supplementary Table S2 and they were similar to those from the first cohort.

### Crude association between treatment and clinical outcomes/death/costs

Table 2 demonstrates the length of observation, the incidence of clinical outcomes, mortality rate and daily healthcare costs by medication groups. The median observation time was shorter for new users of SGLT-2 inhibitors (median observation, 310 days; interquartile range, 90–464 days), but longer for those of DPP-4 inhibitors (median observation, 453 days; interquartile range, 255–488 days). New users of GLP-1 agonists had higher incidence of amputation (6.1 per 10,000 person-years) than those of SGLT-2 inhibitors (1.5 per 10,000 person-years) and DPP-4 inhibitors (4.6 per 10,000 person-years) and users of older agents (1.9 per 10,000 person-years). New users of DPP-4 inhibitors (69.9 per 10,000 person-years) also had higher mortality rate than those of SGLT-2 inhibitors (10.8 per 10,000 person-years) and GLP-1 agonists (18.3 per 10,000 person-years) and users of older agents (52.5 per 10,000 person-years). In addition, new users of GLP-1 agonists (NTD$ 364) had higher total costs per day than those initiating SGLT-2 inhibitors (NTD$ 235), DPP-4 inhibitors (NTD$ 253) and other older medications (NTD$ 174).

**Table 2:** Incidence of amputation and other vascular outcomes, mortality rate and costs per day among new users of SLGT-2 inhibitors, DPP-4 inhibitors, GLP-1 agonists, or other oral treatments for type 2 diabetes.

Cohort	SGLT-2	DPP-4	GLP-1	Other
**First cohort (excluding amputation during baseline)**				
Amputation, No	1	82	1	171
Total No. of person-years	6,507.4	178,188.1	1,634.8	898,430.2
Rate per 10 000 person-years	1.5	4.6	6.1	1.9
Observation time, median (IQR), days	310 (90–464)	453 (255–488)	439.5 (151–480)	422 (116–481)
Death	7	1,246	3	4,713
Total No. of person-years	6,507.9	178,221.1	1,635.0	898,513.5
Rate per 10 000 person-years	10.8	69.9	18.3	52.5
Observation time, median (IQR), days	310 (90–464)	453 (255–488)	439.5 (151–480)	422 (116–481)
Cost				
Total cost per day	235	253	364	174
Emergency cost per day	3	6	6	6
Inpatient cost per day	37	46	24	36
Observation time, median (IQR), days	310 (90–464)	453 (255–488)	439.5 (152–480)	422 (116–481)
**Second cohort (excluding any outcome during baseline)**				
Ulcer, No	10	303	3	1,167
Total No. of person-years	6,294.1	170,468.9	1,577.7	867,221.7
Rate per 10,000 person-years	15.9	17.8	19.0	13.5
Observation time, median (IQR), days	310 (90–464)	453 (254–488)	440 (153–480)	420 (114–481)
Osteomyelitis	7	171	3	708
Total No. of person-years	6,295.2	170,538.1	1,577.0	867,487.5
Rate per 10,000 person-years	11.1	10.0	19.0	8.2
Observation time, median (IQR), days	310 (90–464)	453 (254–488)	440 (154–480)	420 (115–481)
Peripheral vascular disease	71	2,257	16	10,999
Total No. of person-years	6,257.6	169,326.0	1,569.3	861,726.2
Rate per 10,000 person-years	113.5	133.3	102.0	127.6
Observation time, median (IQR), days	304 (90–464)	452 (252–487)	439 (152–480)	415 (112–481)
Critical limb ischemia	0	31	1	97
Total No. of person-years	6,298.4	170,622.8	1,577.7	867,799.1
Rate per 10,000 person-years	0.0	1.8	6.3	1.1
Observation time, median (IQR), days	310 (90–464)	453 (255–488)	440 (154–480)	420 (115–481)

DPP-4, dipeptidyl peptidase 4; GLP-1, glucagon-like peptide 1; IQR, interquartile range; SGLT-2, sodium-glucose cotransporter 2.

### Adjusted association between and clinical outcomes/death/costs

Table 3 shows the adjusted association of diabetes medicines with outcomes of interest. After propensity score weighting and adjusting for potentially confounders, there were statistically significantly lower risks of death associated with new users of SGLT-2 inhibitors compared with new users of DPP-4 inhibitors (adjusted hazard ratio [aHR], 0.27; 95% CIs, 0.12–0.62) or users of first-line agents (aHR, 0.28; 95% CIs, 0.12–0.64), but not compared with new users of GLP-1 agonist (aHR, 0.62; 95% CIs, 0.20–1.97). However, there were mostly non-statistically significant risks across all five clinical outcomes associated with new users of SGLT-2 inhibitors compared with new users of DPP-4 inhibitors and GLP-1 agonists and users of older agents; the only exception was that new users of SGLT-2 inhibitors were more likely to have higher rates of peripheral vascular diseases compared with new users of GLP-1 agonists (aHR, 1.47; 95% CIs, 1.03–2.09).

**Table 3:** Adjusted association between use of SGLT-2 inhibitors and outcomes of interest.

Cohort	DPP-4 inhibitors		GLP-1 agonists		Other drugs	
	HR/Ratio	95%CI	HR/Ratio	95%CI	HR/Ratio	95%CI
**First cohort (excluding amputation during baseline)**						
Amputation	0.38	(0.04–3.73)	−	–	0.61	(0.05–7.36)
Death	0.27	(0.12–0.62)	0.62	(0.20–1.97)	0.28	(0.12–0.64)
**Cost**						
Total cost per day	1.13	(1.12–1.13)	0.64	(0.63–0.66)	1.35	(1.35–1.36)
Emergency cost per day	0.78	(0.76–0.80)	1.05	(0.95–1.17)	0.55	(0.55–0.56)
Inpatient cost per day	0.87	(0.84–0.89)	1.12	(1.07–1.18)	0.84	(0.83–0.85)
**Second cohort (excluding any outcome during baseline)**						
Ulcer	1.20	(0.50–2.88)	1.50	(0.57–3.94)	1.20	(0.49–2.96)
Osteomyelitis	1.27	(0.44–3.65)	0.57	(0.21–1.54)	1.38	(0.46–4.18)
Peripheral vascular disease	1.10	(0.80–1.52)	1.47	(1.03–2.09)	1.01	(0.74–1.40)
Critical limb ischemia	−	–	−	–	−	–

Regressions with propensity score weighting adjusted for demographics (gender and age), diabetes severity, comorbidities (cerebrovascular disease, congestive heart failure, ischemic heart disease, hypertension, retinopathy, nephropathy, neuropathy, atrial fibrillation, renal disease, and eye disease) and medication histories during the baseline period (angiotensin-converting enzyme inhibitors, anticoagulants, angiotensin receptor blockers, aspirin, antiasthmatic drugs, bile acid sequestrants, carbonic anhydrase inhibitors, calcium channel blockers, fibrates, hormone replacement therapy, loop diuretic, β-blockers, platelet aggregation inhibitors, potassium-sparing diuretic, statins, and thiazides).

CI, confidence interval; DPP-4, dipeptidyl peptidase 4; GLP-1, glucagon-like peptide 1; HR, hazard ratio.

For cost outcomes, new users of SGLT-2 inhibitors had lower daily total healthcare costs compared with those of GLP-1 agonists (adjusted cost ratio (aCR), 0.64; 95% CIs, 0.63–0.66), but higher compared with those of DPP-4 inhibitors (aCR, 1.13; 95% CIs, 1.12–1.13) or users of first-line medications (aCR, 1.35; 95% CIs, 1.35–1.36); new users of SGLT-2 inhibitors had lower daily inpatient costs compared with those of DPP-4 inhibitors (aCR, 0.87; 95% CIs, 0.84–0.89) and users of first-line medications (aCR, 0.84; 95% CIs, 0.83–0.85), but higher daily inpatient costs compared with those of GLP-1 agonists (aCR, 1.12; 95% CIs, 1.07–1.18). We also presented the risk-adjusted Kaplan–Meier survival curves in the Supplementary Fig. S1.

## Discussion

In this Taiwanese National Health insurance database, new users of SGLT-2 inhibitors are associated with lower all-cause mortality compared with new users of DPP-4 inhibitor and users of other older agents, and lower total costs compared with new users of GLP-1 agonist. However, there was no difference in the risk of lower-extremity amputations between SGLT-2 inhibitor and other agent classes, namely DPP-4 inhibitor, GLP-1 agonist and other older agents.

The crude risk of lower-extremity amputation among new SGLT-2 inhibitor users in this study differs from previous trials. The CANVAS trial study reported a rate of 6.4 participants per 1000 patient-years for amputation in toes, feet and legs among canagliflozin new users (compared with 3.4 participants per 1000 patient-years among placebo patients)^8^. However, the incidence rate of amputation in this study across all medication groups only ranged between 1.5 to 6.1 participants per 10,000 patient-years. This may attribute to the easy healthcare access and affordable healthcare under Taiwan’s health system. Therefore, patients would not need to wait till the disease becomes very severe to see a doctor.

The CANVAS study concluded an increased risk of amputation caused by canagliflozin use (HR = 1.97; 95% CIs [1.41 to 2.75])^8,33^, which we did not observe in this study. However, a direct comparison of their results with ours might not be appropriate. First, the population within CANVAS study is very different from that of our study. Our study population has much lower prevalence of baseline use of statin (24.3% vs 74.8%), ACE inhibitors/angiotensin receptor blockers (3.5%/13.4% vs 80.2%), beta blocker (12.6% vs 52.4%) and potassium-sparing diuretic (2.2% vs 35.9%) as co-medication across classes of antidiabetic agents, indicating a higher inherent CVD risk within CANVAS population. Furthermore, our study adopted three comparison groups consisting of three different classes of medications while CANVAS trial only had a placebo reference group. Despite this potential incomparability between our study and CANVAS study, the death HRs of all-cause mortality in this study comparing new use of SGLT-2 inhibitor to DPP-4 inhibitor and first-line agents were in the direction similar to the HR of death reported in EMPA-REG OUTCOME trial comparing empagliflozin to placebo (HR = 0.68; 95% CIs, 0.57 to 0.82) and that of CVD-related death reported in CANVAS study comparing canagliflozin to placebo (HR = 0.86; 95% CIs, 0.75 to 0.97)^8,34^.

Our results are in large consistent with some previous observational study. Similar to current findings, a real-world meta-analysis of 4 observational databases found insignificantly decreased hazard of below-knee lower amputation comparing canagliflozin vs non-SGLT2 medications (HR = 0.75; 95% CIs, 0.40 to 1.41)^35^. Our previous analysis using Truven Commercial Claims and Encounters Data and similar methodology suggested no significant increase in risk of foot and leg amputation comparing new SGLT-2 inhibitor use against new DPP-4 inhibitor (HR = 1.50, 95% CIs, 0.85–2.67) and GLP-1 agonist use (HR = 1.47, 95% CIs, 0.64–3.36); the same study found twofold increase in risk of foot and leg amputation when comparing new SGLT-2 inhibitor use against older agents grouped together (HR = 2.12; 95% CIs, 1.19–3.77), while this study did not^3^. The exception comes from the register-based cohort study in Sweden and Denmark, where the researchers identified an increased risk of lower-extremity amputation comparing SGLT-2 inhibitor vs GLP-1 receptor agonists (HR = 2.32; 95%CI 1.37 to 3.91)^36^. However, due to the extremely low incidence rate of amputation in both SGLT-2 inhibitor and GLP-1 agonist users, we were underpowered to conduct any meaningful risk comparison between SGLT-2 inhibitor and GLP-1 agonist in this study.

Studies utilizing real-world data consistently found users of SGLT-2 inhibitors were associated with lower risk of all-cause mortality (relative risks ranging from 0.50 –0.59)^9,12^, and cardiovascular mortality^11^. However, many of these observational studies defined their comparison group as a combination of patients who have never used any type of anti-diabetic medications and previous users of DPP-4 inhibitor, GLP-1 agonist or other older agents. This practice might be problematic as the mortality risk could differ dramatically among (1) patients who switched from other previous medications vs. non-switchers and (2) patient initiating on different classes of anti-diabetes medications due to various reasons (the existence of other chronic conditions at the time of dispense, age, etc.)^37^.

Available cost studies comparing one anti-diabetic drug or class against another defined costs differently from ours. One study that has defined costs closest to ours, i.e. total healthcare cost during baseline and follow-up periods, found a cost ratio of 0.95 (*p* = 0.256) comparing Dapagliflozin cohort against Sitagliptin cohort^14^. In contrast, our results suggested SGTL-2 is significantly more expensive than DPP-4 inhibitor and older agents but cheaper than GLP-1 agonist. This could be explained by the differing prescription patterns and clinical guideline associated with treating diabetes in US vs Taiwan.

This study was one of the first studies to assess the effectiveness, safety and costs associated with all available classes of anti-diabetes agents within the same population, which put relative risk or cost ratio measures under the same framework of reference. Second, we used an active comparator new user design that helps reduce the bias by indication and healthy user bias^37^. Third, switchers during the follow-up period were properly censored. Fourth, covariates based on literature reviews were adjusted as confounders^3,35,38^. In addition, the study avoided grouping together medications that belong to different agents or classes (such as grouping GLP-1 agonist and DPP-4 inhibitor into one reference group), thus provides pharmacologically sound effect estimates.

Our study has limitations. First, our investigation into the foot and leg amputation risk comparing SGLT-2 inhibitor and GLP-1 agonist in both cohorts is insufficiently powered due to extremely small amount of cases in both new user populations. Given the data availability we could only observe the participants for 1 year and 5 months; even though the observation was not long, it was still between three to four times longer than that in a similar study using American claims data^3^. Second, the conclusion based on NHIRD might have limited external validity for other populations due to differences in clinical practice and health systems. For example, traditional Chinese medicine is reimbursable in the treatment of diabetes and other comorbidities under Taiwan national health insurance system.

Last, as observational studies, even though we attempted to adjust for confounding based on prior research^3,35^ and propensity score weighting, there might still be bias in our estimates due to the unmeasured confounding. For example, potential residual confounding that may partially explain the observed reduced risk of death associated with SGLT-2 inhibitors use might include other comorbidities (such as cancer, chronic lung and liver disease, and dementia), patient socio-economic status, physician characteristics, and regional characteristics. Nevertheless, under Taiwan’s national healthcare system where all of these medications are reimbursed and the pharmaceutical copayment is low, decision to treat with certain medications are less driven by patient socio-economic status, physician characteristics, and regional characteristics than clinical indications, which were rigorously controlled for in our statistical model with propensity score weighting. Before propensity score weighting, the DPP-4 users were older, and had severe diabetes and more comorbidities. According to Taiwan’s clinical guidelines for diabetes care, DPP-4 inhibitors have fewer side effects, including fractures, than other newer drugs^39^. In addition, DPP-4 inhibitors has been reimbursed since March 1, 2009. Therefore, physicians are more likely to prescribe DPP-4 inhibitors for patients who were older, and had severe diabetes and more comorbidities to avoid side effects. Moreover, because of lack of cost related to the management of diabetes and complications among the treatment groups, we could not compare the differences in cost among the treatment groups.

Given the differing effectiveness, safety and cost across classes of anti-diabetes medications, future studies should focus on the cost–benefit and cost-safety associated with the use of different anti-diabetes drugs or classes of such drugs. Future studies may also investigate potential factors that explain the dramatic effect estimates under different study design as well as data sources (such as RCT vs observational, or register-based vs claims data-based study).

Our study suggested that SGLT-2 inhibitors use is associated with lower risk of mortality compared to the use of DPP 4 inhibitors and lower costs compared to the use of GLP-1 agonists. Healthcare providers should consider the benefits and drawbacks when prescribing the newer agents for diabetic patients.

## Supplementary Information

Supplementary Information

## Data Availability

The data that support the findings of this study are available from NHIA but restrictions apply to the availability of these data, which were used under license for the current study, and so are not publicly available. Data are however available from the authors upon reasonable request and with permission of the NHIA.
