# Supplementary material for: Association of antidiabetic therapies with lower extremity amputation, mortality and healthcare cost from a nationwide retrospective cohort study in Taiwan
Source: Sci Rep. 2021 Mar 26;11:7000. doi: 10.1038/s41598-021-86516-4 (PMC7997872; doi:10.1038/s41598-021-86516-4)

**Association of antidiabetic therapies with lower extremity amputation, mortality and healthcare cost from a nationwide retrospective cohort study in Taiwan**

Hsien-Yen Chang, PhD^1,2,3^

Ying-Yi Chou, PhD^4^

Wenze Tang, MPH^5^

Guann-Ming Chang, MD^6^

Chi‐Feng Hsieh, PhD^7^

Sonal Singh, MD, MPH^8^

Yu-Chi Tung, PhD^4^

^1^ Department of Health Policy and Management, Johns Hopkins Bloomberg School of Public Health, Baltimore, Maryland

^2^ Center for Population Health IT, Johns Hopkins Bloomberg School of Public Health, Baltimore, Maryland

^3^ Center for Drug Safety and Effectiveness, Johns Hopkins Bloomberg School of Public Health, Baltimore, Maryland

^4^ Institute of Health Policy and Management, School of Public Health, National Taiwan University, Taipei, Taiwan

^5^ Department of Epidemiology, Harvard School of Public Health, Boston, Massachusetts

^6^Department of Family Medicine, Chang-Gung Memorial Hospital, Taoyuan, Taiwan

^7^ School of Medicine for International Students, I- Shou University, Kaohsiung, Taiwan

^8^ Department of Family Medicine and Community Health, University of Massachusetts Medical School, Worcester, Massachusetts

Address for correspondence: Yu-Chi Tung

Professor

Institute of Health Policy and Management,

School of Public Health,

National Taiwan University,

Room 634, No.17, Xu-Zhou Road,

Taipei 100, Taiwan,

Tel: 886-2-33668064;

Fax: 886-2-33668064;

Email: [yuchitung@ntu.edu.tw](mailto:yuchitung@ntu.edu.tw)

**Appendix. Outcomes**

**Amputation:**

**Definition**

**ICD‐9‐CM procedure code:**

**84.11**

**84.12**

**84.13**

**84.14**

**84.15**

**84.16**

**84.17**

**ICD‐10 procedure code:**

**0Y6.x**

**Source**

1. **Newton KM, Wagner EH, Ramsey SD, McCulloch D, Evans R, Sandhu N, Davis C. The use of automated data to identify complications and comorbidities of diabetes: a validation study. J Clin Epidemiol. 1999;52(3):199–207.**
2. **Chang HY, Singh S, Mansour O, Baksh S, Alexander GC. Association between sodium-glucose cotransporter 2 inhibitors and lower extremity amputation among patients with type 2 diabetes. JAMA Intern Med. 2018;178(9):1190-8.**
3. **Agency for Healthcare Research and Quality. Lower-extremity amputation among patients with diabetes rate 2020 [Available from: https://www.qualityindicators.ahrq.gov/Modules/PQI_TechSpec_ICD10_v2020.aspx.**

**Ulcer:**

**Definition**

**ICD‐9‐CM: 707.1x**

**ICD‐10: L97.x**

**Positive Predictive Value: 88.5**

**Source**

1. **Newton KM, Wagner EH, Ramsey SD, McCulloch D, Evans R, Sandhu N, Davis C. The use of automated data to identify complications and comorbidities of diabetes: a validation study. J Clin Epidemiol. 1999;52(3):199–207.**
2. **Chang HY, Singh S, Mansour O, Baksh S, Alexander GC. Association between sodium-glucose cotransporter 2 inhibitors and lower extremity amputation among patients with type 2 diabetes. JAMA Intern Med. 2018;178(9):1190-8.**
3. **Romon I, Jougla E, Balkau B, Fagot-Campagna A. The burden of diabetes-related mortality in France in 2002: an analysis using both underlying and multiple causes of death. Eur J Epidemiol. 2008;23(5):327-34.**

**Osteomyelitis:**

**Definition**

**ICD‐9‐CM: 730.xx**

**ICD‐10: M86.x**

**Positive Predictive Value: 63.9**

**Source**

1. **Newton KM, Wagner EH, Ramsey SD, McCulloch D, Evans R, Sandhu N, Davis C. The use of automated data to identify complications and comorbidities of diabetes: a validation study. J Clin Epidemiol. 1999;52(3):199–207.**
2. **Chang HY, Singh S, Mansour O, Baksh S, Alexander GC. Association between sodium-glucose cotransporter 2 inhibitors and lower extremity amputation among patients with type 2 diabetes. JAMA Intern Med. 2018;178(9):1190-8.**
3. **Romon I, Jougla E, Balkau B, Fagot-Campagna A. The burden of diabetes-related mortality in France in 2002: an analysis using both underlying and multiple causes of death. Eur J Epidemiol. 2008;23(5):327-34.**

**Peripheral vascular disease:**

**Definition**

**ICD‐9‐CM:**

**093.0**

**437.3**

**440.xx**

**441.xx**

**443.1x-443.9x**

**47.1x**

**557.1x**

**557.9x**

**V43.4x**

**ICD‐10:**

**I70.x**

**I71.x**

**I73.1x**

**I73.8x**

**I73.9x**

**I77.1x**

**I79.0x**

**I79.2x**

**K55.1x**

**K55.8x**

**K55.9x**

**Z95.8x**

**Z95.9x**

**Source**

**Quan H, Sundararajan V, Halfon P, Fong A, Burnand B, Luthi JC, et al. Coding algorithms for defining comorbidities in ICD-9-CM and ICD-10 administrative data. Med Care. 2005;43(11):1130-9.**

**Critical limb ischemia**

**Definition**

**ICD‐9‐CM:**

**440.22**

**440.23**

**440.24**

**443.9x**

**ICD‐10:**

**I70.22**

**I70.23**

**I70.24**

**Source**

1. **Bekwelem W, Bengtson LG, Oldenburg NC, Winden TJ, Keo HH, Hirsch AT, Duval S. Development of administrative data algorithms to identify patients with critical limb ischemia. Vasc Med. 2014;19:483–490.**
2. **Chang HY, Singh S, Mansour O, Baksh S, Alexander GC. Association between sodium-glucose cotransporter 2 inhibitors and lower extremity amputation among patients with type 2 diabetes. JAMA Intern Med. 2018;178(9):1190-8.**
3. **Reinecke H, Unrath M, Freisinger E, Bunzemeier H, Meyborg M, Lüders F, et al. Peripheral arterial disease and critical limb ischaemia: still poor outcomes and lack of guideline adherence. Eur Heart J. 2015;36(15):932-8.**

**Supplementary Table S1. Standardized differences between groups before and after the propensity score weighting**

| **Characteristic** | **Before weighting** | | | **After weighting** | | |
| --- | --- | --- | --- | --- | --- | --- |
|  | **SGLT‐2**  **vs.**  **DPP-4** | **SGLT‐2 vs.**  **GLP-1** | **SGLT‐2**  **vs.**  **Other** | **SGLT‐2**  **vs.**  **DPP-4** | **SGLT‐2**  **vs.**  **GLP-1** | **SGLT‐2**  **vs.**  **Other** |
| **Female, No. (%)** | **-0.003** | **-0.163** | **-0.029** | **0.002** | **-0.009** | **0.000** |
| **Age, No. (%)** |  |  |  |  |  |  |
| **18-34 y** | **0.232** | **-0.077** | **0.093** | **0.000** | **0.002** | **0.000** |
| **35-44 y** | **0.348** | **-0.153** | **0.225** | **0.001** | **0.005** | **0.000** |
| **45-54 y** | **0.293** | **-0.069** | **0.182** | **0.001** | **-0.001** | **0.000** |
| **55-64 y** | **0.059** | **0.112** | **0.036** | **-0.001** | **0.013** | **0.000** |
| **≧65 y** | **-0.595** | **0.129** | **-0.385** | **0.000** | **-0.018** | **0.000** |
| **aDCSI score, No. (%)** |  |  |  |  |  |  |
| **0** | **0.157** | **0.056** | **-0.235** | **0.000** | **0.000** | **0.000** |
| **1** | **0.056** | **-0.010** | **0.111** | **-0.001** | **0.009** | **0.000** |
| **2** | **-0.056** | **0.046** | **0.107** | **0.001** | **0.002** | **0.000** |
| **≧3** | **-0.216** | **-0.117** | **0.093** | **0.000** | **-0.014** | **0.000** |
| **Baseline comorbidities, No. (%)** |  |  |  |  |  |  |
| **Cerebrovascular disease** | **-0.195** | **0.051** | **-0.099** | **0.000** | **0.003** | **0.000** |
| **Congestive heart failure** | **-0.047** | **0.078** | **0.065** | **0.004** | **-0.004** | **0.000** |
| **Ischemic heart disease** | **-0.085** | **0.138** | **0.079** | **0.001** | **-0.005** | **0.000** |
| **Hypertension** | **-0.153** | **-0.022** | **-0.009** | **0.001** | **-0.013** | **0.000** |
| **Retinopathy** | **-0.095** | **-0.124** | **0.142** | **-0.003** | **-0.003** | **0.000** |
| **Nephropathy** | **-0.265** | **-0.145** | **0.030** | **-0.002** | **-0.012** | **0.000** |
| **Neuropathy** | **-0.082** | **-0.058** | **0.061** | **-0.002** | **-0.004** | **0.000** |
| **Atrial fibrillation** | **-0.059** | **0.027** | **-0.004** | **0.001** | **0.009** | **0.000** |
| **Renal disease** | **-0.276** | **-0.140** | **0.022** | **-0.002** | **-0.013** | **0.000** |
| **Eye disease** | **-0.050** | **-0.114** | **0.168** | **-0.004** | **-0.010** | **0.000** |
| **Baseline medications, No. (%)** |  |  |  |  |  |  |
| **ACE inhibitors** | **0.043** | **0.058** | **0.002** | **0.001** | **0.001** | **0.000** |
| **Anticoagulants** | **0.029** | **0.098** | **-0.018** | **0.001** | **0.006** | **0.000** |
| **Angiotensin receptor blockers** | **0.085** | **0.078** | **0.005** | **0.000** | **0.000** | **0.000** |
| **Aspirin** | **0.033** | **0.098** | **-0.019** | **0.001** | **0.005** | **0.000** |
| **Asthma** | **0.067** | **-0.039** | **0.019** | **0.001** | **-0.009** | **0.000** |
| **Bile acid sequestrants** | **-0.010** | **0.016** | **-0.005** | **0.000** | **0.016** | **0.000** |
| **Carbonic anhydrase inhibitors** | **-0.042** | **-0.014** | **0.006** | **0.000** | **-0.006** | **0.000** |
| **Calcium channel blockers** | **0.016** | **0.094** | **-0.083** | **0.001** | **0.001** | **0.000** |
| **Fibrates** | **0.041** | **0.117** | **0.003** | **0.000** | **-0.010** | **0.000** |
| **Hormone replacement therapy** | **0.086** | **-0.079** | **-0.014** | **-0.001** | **-0.004** | **0.000** |
| **Loop diuretic** | **-0.040** | **0.028** | **0.042** | **0.002** | **-0.010** | **0.000** |
| **β-Blockers** | **0.106** | **0.110** | **0.011** | **0.003** | **-0.002** | **0.000** |
| **Platelet aggregation inhibitors** | **0.049** | **0.086** | **-0.047** | **0.001** | **0.004** | **0.000** |
| **Potassium-sparing diuretic** | **0.030** | **0.046** | **0.055** | **0.000** | **-0.014** | **0.000** |
| **Statin** | **0.148** | **0.227** | **-0.028** | **0.002** | **-0.003** | **0.000** |
| **Thiazide** | **0.009** | **0.052** | **0.001** | **-0.001** | **-0.026** | **0.000** |

**ACE, angiotensin-converting enzyme; aDCSI, adapted Diabetes Complications Severity Index; DPP-4, dipeptidyl peptidase 4; GLP-1, glucagon-like peptide 1; SD, standard deviation; SGLT-2, sodium-glucose cotransporter 2.**

Supplementary Table S**2**. Characteristics of the study sample from second cohort

| Characteristic | SGLT-2  (n=8,019) | | DPP-4  (n=167,065) | | GLP-1  (n=1,718) | | Other  (n=1,003,984) | | P |
| --- | --- | --- | --- | --- | --- | --- | --- | --- | --- |
| Female, No. (%) | 4,245 | 52.9 | 88,456 | 52.9 | 773 | 45.0 | 519,062 | 51.7 | <0.001 |
| Age, mean (SD) | 56.1 | 12.0 | 65.1 | 11.8 | 53.4 | 12.1 | 61.7 | 12.8 | <0.001 |
| Age, No. (%) |  |  |  |  |  |  |  |  | <0.001 |
| 18-34 y | 365 | 4.6 | 1,334 | 0.8 | 109 | 6.3 | 27,991 | 2.8 |  |
| 35-44 y | 1,092 | 13.6 | 6,523 | 3.9 | 329 | 19.2 | 68,567 | 6.8 |  |
| 45-54 y | 2,048 | 25.5 | 23,508 | 14.1 | 492 | 28.6 | 181,672 | 18.1 |  |
| 55-64 y | 2,697 | 33.6 | 52,166 | 31.2 | 493 | 28.7 | 323,500 | 32.2 |  |
| ≧65 y | 1,817 | 22.7 | 83,534 | 50.0 | 295 | 17.2 | 402,254 | 40.1 |  |
| aDCSI score, No. (%) |  |  |  |  |  |  |  |  |  |
| Mean | 1.0 | 1.2 | 1.3 | 1.4 | 1.1 | 1.3 | 0.8 | 1.1 | <0.001 |
| 0 | 3,450 | 43.0 | 59,441 | 35.6 | 696 | 40.5 | 552,814 | 55.1 | <0.001 |
| 1 | 2,263 | 28.2 | 43,165 | 25.8 | 496 | 28.9 | 229,972 | 22.9 |  |
| 2 | 1,441 | 18.0 | 34,306 | 20.5 | 284 | 16.5 | 140,097 | 14.0 |  |
| ≧3 | 865 | 10.8 | 30,153 | 18.1 | 242 | 14.1 | 81,101 | 8.0 |  |
| Baseline use of antidiabetes medication,  No. (%) |  |  |  |  |  |  |  |  |  |
| Biguanides (Metformin) | 1,410 | 17.6 | 15,033 | 9.0 | 192 | 11.2 | 237,684 | 23.7 | <0.001 |
| Sulfonylureas | 1,277 | 15.9 | 15,115 | 9.0 | 175 | 10.2 | 139,103 | 13.9 | <0.001 |
| Meglitinide | 802 | 10.0 | 6,995 | 4.2 | 82 | 4.8 | 36,271 | 3.6 | <0.001 |
| α-glucosidase inhibitor | 768 | 9.6 | 9,743 | 5.8 | 106 | 6.2 | 41,412 | 4.1 | <0.001 |
| Combinations | 1,875 | 23.4 | 9,453 | 5.7 | 174 | 10.1 | 99,733 | 9.9 | <0.001 |
| Other drugs | 179 | 2.2 | 4,421 | 2.6 | 49 | 2.9 | 19,530 | 1.9 | <0.001 |
| Baseline comorbidities, No. (%) |  |  |  |  |  |  |  |  |  |
| Cerebrovascular disease | 544 | 6.8 | 20,949 | 12.5 | 96 | 5.6 | 95,404 | 9.5 | <0.001 |
| Congestive heart failure | 441 | 5.5 | 11,019 | 6.6 | 68 | 4.0 | 41,544 | 4.1 | <0.001 |
| Ischemic heart disease | 1,432 | 17.9 | 35,376 | 21.2 | 224 | 13.0 | 149,657 | 14.9 | <0.001 |
| Hypertension | 5,264 | 65.6 | 120,859 | 72.3 | 1,134 | 66.0 | 659,694 | 65.7 | <0.001 |
| Retinopathy | 1,088 | 13.6 | 27,991 | 16.8 | 308 | 17.9 | 90,127 | 9.0 | <0.001 |
| Nephropathy | 650 | 8.1 | 27,400 | 16.4 | 209 | 12.2 | 71,959 | 7.2 | <0.001 |
| Neuropathy | 478 | 6.0 | 13,381 | 8.0 | 124 | 7.2 | 45,951 | 4.6 | <0.001 |
| Atrial fibrillation | 128 | 1.6 | 3,971 | 2.4 | 22 | 1.3 | 16,276 | 1.6 | <0.001 |
| Renal disease | 663 | 8.3 | 28,573 | 17.1 | 212 | 12.3 | 76,361 | 7.6 | <0.001 |
| Eye disease | 908 | 11.3 | 21,343 | 12.8 | 257 | 15.0 | 65,121 | 6.5 | <0.001 |
| Baseline medications, No. (%) |  |  |  |  |  |  |  |  |  |
| ACE inhibitors | 274 | 3.4 | 4,491 | 2.7 | 44 | 2.6 | 34,550 | 3.4 | <0.001 |
| Anticoagulants | 792 | 9.9 | 14,914 | 8.9 | 124 | 7.2 | 103,689 | 10.3 | <0.001 |
| Angiotensin receptor blockers | 1,070 | 13.3 | 17,436 | 10.4 | 184 | 10.7 | 131,710 | 13.1 | <0.001 |
| Aspirin | 701 | 8.7 | 12,912 | 7.7 | 107 | 6.2 | 92,256 | 9.2 | <0.001 |
| Asthma | 3,112 | 38.8 | 59,555 | 35.6 | 695 | 40.5 | 381,028 | 38.0 | <0.001 |
| Bile acid sequestrants | 0 | 0.0 | 43 | 0.0 | 0 | 0.0 | 184 | 0.0 | 0.113 |
| Carbonic anhydrase inhibitors | 126 | 1.6 | 3,565 | 2.1 | 31 | 1.8 | 15,179 | 1.5 | <0.001 |
| Calcium channel blockers | 1,067 | 13.3 | 20,976 | 12.6 | 176 | 10.2 | 161,981 | 16.1 | <0.001 |
| Fibrates | 567 | 7.1 | 10,242 | 6.1 | 75 | 4.4 | 70,804 | 7.1 | <0.001 |
| Hormone replacement therapy | 257 | 3.2 | 3,028 | 1.8 | 81 | 4.7 | 34,525 | 3.4 | <0.001 |
| Loop diuretic | 436 | 5.4 | 10,696 | 6.4 | 84 | 4.9 | 46,158 | 4.6 | <0.001 |
| β-Blockers | 993 | 12.4 | 15,159 | 9.1 | 155 | 9.0 | 121,361 | 12.1 | <0.001 |
| Platelet aggregation inhibitors | 1,527 | 19.0 | 28,433 | 17.0 | 268 | 15.6 | 208,510 | 20.8 | <0.001 |
| Potassium-sparing diuretic | 168 | 2.1 | 2,854 | 1.7 | 26 | 1.5 | 14,133 | 1.4 | <0.001 |
| Statin | 1,950 | 24.3 | 30,442 | 18.2 | 262 | 15.3 | 255,647 | 25.5 | <0.001 |
| Thiazide | 286 | 3.6 | 5,789 | 3.5 | 44 | 2.6 | 36,405 | 3.6 | 0.001 |

ACE, angiotensin-converting enzyme; aDCSI, adapted Diabetes Complications Severity Index; DPP-4, dipeptidyl peptidase 4; GLP-1, glucagon-like peptide 1; SD, standard deviation; SGLT-2, sodium-glucose cotransporter 2.


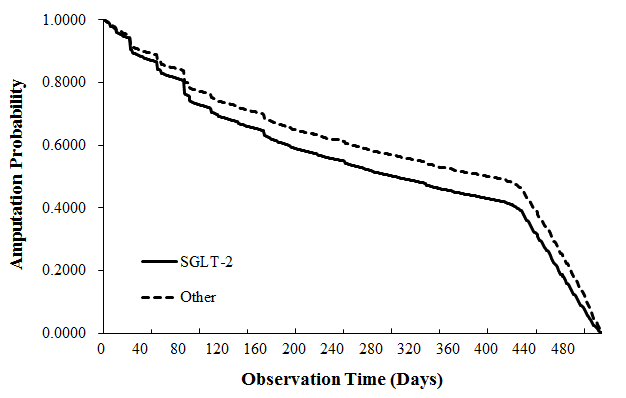

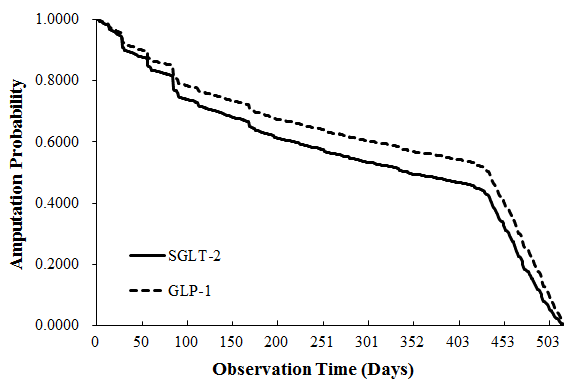
Supplementary Figure S1. Risk-adjusted Kaplan-Meier survival curve


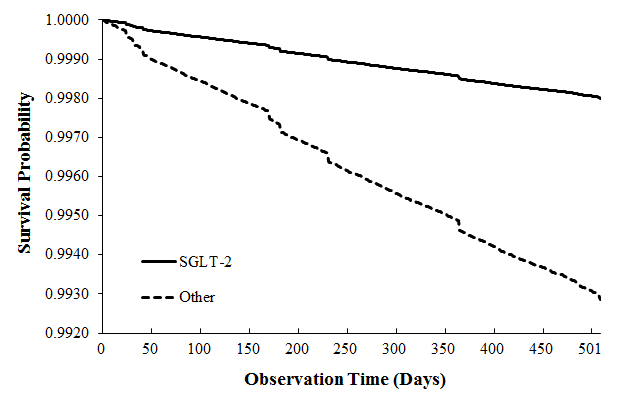

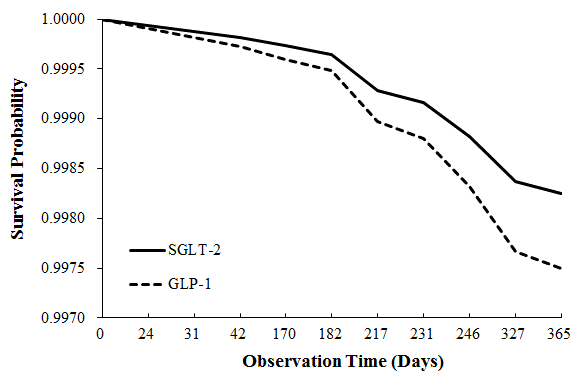

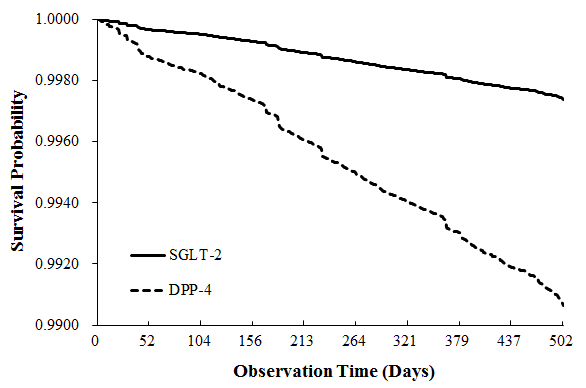

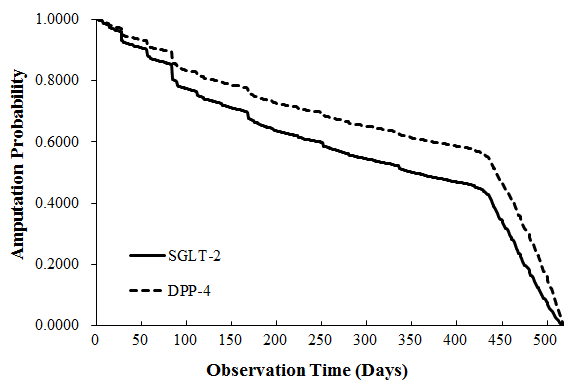

Supplement: Supplementary file 1 — Supplementary Information [file 41598_2021_86516_MOESM1_ESM.docx]
